# Vitamin C suppresses cell death in MCF-7 human breast cancer cells induced by tamoxifen

**DOI:** 10.1111/jcmm.12188

**Published:** 2013-11-25

**Authors:** Tamilselvan Subramani, Swee Keong Yeap, Wan Yang Ho, Chai Ling Ho, Abdul Rahman Omar, Suraini Abdul Aziz, Nik Mohd Afizan Nik Abd Rahman, Noorjahan Banu Alitheen

**Affiliations:** a Department of Cell and Molecular Biology, Faculty of Biotechnology and Biomolecular Sciences, Universiti Putra MalaysiaSerdang, Selangor, Malaysia; b Institute of Bioscience, Universiti Putra MalaysiaSerdang, Malaysia; c Department of Bioprocess Technology, Faculty of Biotechnology and Biomolecular Sciences, Universiti Putra MalaysiaSerdang, Selangor, Malaysia

**Keywords:** vitamin C, tamoxifen, breast cancer, ROS, free radical, apoptosis, lipid peroxidation

## Abstract

Vitamin C is generally thought to enhance immunity and is widely taken as a supplement especially during cancer treatment. Tamoxifen (TAM) has both cytostatic and cytotoxic properties for breast cancer. TAM engaged mitochondrial oestrogen receptor beta in MCF-7 cells and induces apoptosis by activation of pro-caspase-8 followed by downstream events, including an increase in reactive oxygen species and the release of pro-apoptotic factors from the mitochondria. In addition to that, TAM binds with high affinity to the microsomal anti-oestrogen-binding site and inhibits cholesterol esterification at therapeutic doses. This study aimed to investigate the role of vitamin C in TAM-mediated apoptosis. Cells were loaded with vitamin C by exposure to dehydroascorbic acid, thereby circumventing *in vitro* artefacts associated with the poor transport and pro-oxidant effects of ascorbic acid. Pre-treatment with vitamin C caused a dose-dependent attenuation of cytotoxicity, as measured by acridine-orange/propidium iodide (AO/PI) and Annexin V assay after treatment with TAM. Vitamin C dose-dependently protected cancer cells against lipid peroxidation caused by TAM treatment. By real-time PCR analysis, an impressive increase in FasL and tumour necrosis factor-α (TNF-α) mRNA was detected after TAM treatment. In addition, a decrease in mitochondrial transmembrane potential was observed. These results support the hypothesis that vitamin C supplementation during cancer treatment may detrimentally affect therapeutic response.

## Introduction

Tamoxifen (TAM) is commonly administered to oestrogen receptor (ER) positive breast cancer patients 1. In such cases, TAM binds the ER to induce apoptosis by preventing oestrogen binding, which normally activates pro-survival signalling pathways. However, other studies have provided evidence of ER-independent mechanisms of apoptotic induction by TAM 2,3. The anti-tumour activity of TAM is believed to be both cytostatic and cytotoxic for breast cancer. More specifically, TAM has been shown to interact with the mitochondrial ER and increasing reactive oxygen species (ROS) concentrations from the mitochondria that were required for cytotoxicity 4,5. Tamoxifen exhibits a complex pharmacology and some additional targets were identified that could account for its anti-cancer activity and may ultimately be involved in treatment failure and resistance 6. At the molecular level, TAM has been shown to modulate cholesterol metabolism through its interaction with the microsomal anti-oestrogen-binding site 7–9. Currently, several cancer patients combine some forms of complementary and alternative medicines with their conventional therapies. The most common choice of these therapies is the use of antioxidant such as vitamin C. In many cases, cancer therapy produces ROS which attack healthy cells and tissues, consequently leading to further damage and unintentional side effects. These adverse effects may be decreased by antioxidants, but it must be assumed that any antioxidant found to reduce toxicity of tumour therapy on healthy tissue has the potential to decrease effectiveness of cancer therapy on malignant cells. Vitamin C possesses proactive role against some types of cancer and also demonstrated the immunomodulatory effects enhancing host defence 10–12. Generally vitamin C is considered supportive for immune responsiveness, and supplementation is common. Vitamin C is transported in the form of dehydroxy ascorbic acid (DHA) among various cells through glucose transporters 13,14. Increased uptake and metabolism of glucose are characteristic of malignant transformation and the overexpression of glucose transporters is a common event in malignancies 15. Therefore, tumour cells often accumulate vitamin C greater to normal cells and may be better protected against the negative effects of ROS. Here, we analysed the protective ability of vitamin C on a tumour treatment modality which is based merely on the formation of ROS. Earlier studies reported that the antioxidant vitamin E completely blocked the effects of microsomal anti-oestrogen-binding site ligands by protecting sterols against oxidation, indicating that ROS were important in these effects 16,17. In this study, we investigated whether vitamin C interferes with TAM treatment on breast cancer cells. We analysed the impact of vitamin C on the induction of oxidative cell injury, therefore, changes in lipid peroxidation and mitochondrial membrane potential were measured, the role of apoptosis-relevant pathways was examined, and the survival rate of MCF-7 human breast cancer cells on treatment was determined.

## Materials and methods

### Cell culture

MCF-7 cells, a human breast cancer cell line obtained from the American Type Culture Collection (Rockville, MD, USA), were cultured until passage 30. Cells were maintained as monolayers in RPMI 1640 medium supplemented with 2 g/l sodium bicarbonate, 1.2 mmol/l glutamine (pH 7.4), 10% (vol/vol) foetal bovine serum, 100 U/ml penicillin and 100 μg/ml streptomycin at 37°C in a humidified environment containing 5% CO_2_.

### Vitamin C treatment

Cells were loaded with vitamin C by exposure to DHA, thereby circumventing *in vitro* artefacts associated with the poor transport and pro-oxidant effects of ascorbic acid 18,19. Vitamin C in the form of DHA is transported through facilitative glucose transporters. Therefore, freshly prepared DHA solution in RPMI 1640 medium was added to MCF-7 cells to achieve 50 and 500 μM final concentrations. As a standard procedure, MCF-7 cells were incubated with vitamin C for 30 min. at 37°C before TAM treatment.

### Determination of vitamin C in MCF-7 cells

Vitamin C uptake was measured as intracellular accumulation after incubation of cells with DHA. Cells were washed in PBS and 1 × 10^6^ cells were lysed in 70 μl 4% phosphoric acid, and centrifuged at 13,000 × *g* for 1 min. at 4°C. The supernatant was transferred into a fresh tube and quantified using a colorimetric assay as previously described 20,21. Briefly, 25 μl of the supernatant was mixed with 10 μl of potassium phosphate buffer (0.1 mol/l, pH 6.5) and 200 μl 4-hydroxy-2,2,6,6-tetramethylpiperidinyloxy free radical (2 mg Tempol per 10 ml of phosphate buffer). After an incubation time of 2 min., 85 μl of *o*-diphenylamine (5 mg per 10 ml of phosphate buffer and 10 μl of potassium phosphate buffer (0.1 mol/l; pH 6.5) were added. The formation of the coloured product was measured at 340 nm and compared with the concentration of a known standard (50 μM vitamin C/L).

### Cell viability analysis

Tamoxifen-induced cell death in DHA-treated MCF-7 cells was quantified using propidium iodide (PI) and acridine-orange (AO) double staining according to standard procedures and examined under fluorescence microscope (Bio-Rad, Hercules, CA, USA). Briefly, treatment was carried out in a 25 ml culture flask. MCF-7 cells were plated at concentration of 1 × 10^6^ cell/ml, and treated with DHA at 50 and 500 μM concentrations for 30 min. The cells were then treated with TAM at 9.5 ± 0.27 μg/ml (IC_50_). Flasks were incubated in atmosphere of 5% CO_2_ at 37°C for 24 hrs. The cells were then spun down at 1000 × *g* for 10 min. Supernatant was discarded and the cells were washed twice using PBS after centrifuging at 1000 × *g* for 10 min. to remove the remaining media. Ten microlitres of fluorescent dyes containing AO (10 μg/ml) and PI (10 μg/ml) was added into the cellular pellet at equal volumes of each. Freshly stained cell suspension was dropped into a glass slide and covered by coverslip. Slides were observed under UV-fluorescence microscope within 30 min. before the fluorescence colour starts to fade. All the treatments and time-point were carried out in three individual experiments. Acridine-orange and PI are intercalating nucleic acid specific fluorochromes which emit green and orange fluorescences, respectively, when they are bound to DNA. Of the two, only AO can cross the plasma membrane of viable and early apoptotic cells. Viewed by fluorescence microscopy, viable cells appear to have green nucleus with intact structure while apoptotic cells exhibit a bright-green nucleus showing condensation of chromatin as dense green areas. Late apoptotic cells and necrotic cells will stain with both AO and PI. Comparatively, PI produces the highest intensity emission. Hence, late apoptotic cells exhibited an orange nucleus showing condensation of chromatin whilst necrotic cells display an orange nucleus with intact structure.

### Assessment of apoptosis

Cells were double stained with annexin V-Fluos and PI and apoptosis was evaluated by fluorescence-activated cell sorting analysis. Annexin V-Fluos was used in accordance with the manufacturer's instructions. Briefly, the cells were harvested, washed in PBS and suspended in annexin V-Fluos labelling solution (10 mM Hepes/NaOH, pH 7.4; 140 mM NaCl, 5 mM CaCl_2_) with PI (1 μg/ml). The suspension was incubated at room temperature for 10 min. and analysed using the BD FACSCanto flow cytometry system. Cells were gated on the basis of their forward and side light scatter, with cell debris excluded from analysis. Data from 10,000 cells/sample were analysed using dedicated software (Bio-Rad). Cells exhibiting positive staining with annexin V (*i.e*. early apoptotic cells exhibiting annexin V+/PI− and late apoptotic/secondary necrotic cells exhibiting Annexin V+/PI+) were considered apoptotic. A two-way dot plot was prepared to verify the percentage of apoptotic cells. Annexin V−/PI− cells were used as viable cell control, and Annexin V−/PI+ cells were considered necrotic.

### Assessment of lipid peroxidation and ROS

High amounts of reactive oxygen intermediates result in lipid peroxidation. Therefore, analysis of malondialdehyde as a marker of lipid peroxidation end products was carried out as described previously 22,23. Approximately 3 × 10^6^ cells were washed in ice-cold PBS, lysed in 260 μl solubilization buffer [10 mM Tris, pH 7.4; 9 g/l NP40; 1 g/l SDS and 250 U/ml benzonase] and centrifuged at 20000 × *g* for 10 min. at 4°C. For protein measurement, an aliquot of 50 μl was frozen at −20°C. The amount of 200 μl of cell lysate or malondialdehyde standards were mixed with 10 μl butylated hydroxytoluene (50 mg/ml ethanol) and 200 μl of orthophosphoric acid (0.2 mM). The reaction mixture incubated on ice for 30 min. then spin down at 2000 × *g* for 15 min. at 25°C. Thereafter, 25 μl of 2-thiobarbituric acid reagent (800 mg of 2-thiobarbituric acid dissolved in 50 ml of 0.1 M NaOH) was added to the supernatant and incubated at 90°C for 45 min. Formed malondialdehyde equivalents, thiobarbituric acid-reactive substances (TBARS) were extracted and measured using a plate reader (Bio-Rad) with excitation at 532 and 600 nm. For quantitative determination of TBARS, 200 μl of a malondialdehyde standard solution was used instead of cell lysate. For this, 50 μl of 1,1,3,3,-tetramethoxypropane (10 mM) was hydrolyzed in 10 ml of 0.01 M HCl for 10 min. at room temperature and then diluted with ultrapure water to suitable concentrations. The protein content was measured spectrophotometrically by Bradford assay. The superoxide dismutase (SOD) activities in the treated cells were measured as previously described 24. Briefly, the cells were harvested and the cell lysate were prepared by above described method. The amount of 100 μl of cell lysate or SOD standards was mixed with 200 μl of master mix (NaCN – 1.5 mg/ml of NaOH; NBT – 1.22 mg/ml of ethanol; Riboflovin – 0.4516 mg/ml distilled water; 0.1 M phosphate buffer, pH: 7.8) in 96 well plates. The reaction mixture was measured using a plate reader (Bio-Rad) at 560 nm in dark. For quantitative determination of SOD, 100 μl of 5 mM vitamin C solution was used instead of cell lysate.

### Total RNA preparation and quantitative Real-Time PCR analysis

The cells were trypsinized and washed twice with PBS. Total RNA was prepared using a Qiagen RNA extraction kit. The RNA concentration was determined by reading the absorbance at 260 and 280 nm with a UV spectrophotometer. A total of 2 μg of cDNA was synthesized according to the manufacturer's instructions (SuperScript III First-Strand Synthesis System). The quantitative real-time PCR was performed with the Bio-Rad Real-Time PCR detection system and the QuantiTect SYBR Green PCR kit (Qiagen, Kuala Lumpur, Malaysia) according to the manufacturer's suggestions. Glyceraldehyde 3-phosphate dehydrogenase (GAPDH) mRNA levels were also quantified in each sample and were used as a normalization control. The following primer pairs were used: 5′-CAGATGGGCTGTACCTTATC-3′ and 5′-GGACTCCGTGATGTCTTAGTA-3′ (TNF-α); 5′-TCTGGAATGGGAAGACACATA-3′ and 5′-ACCAGATCCCCAGGATACTT-3′ (FasL) and 5′-GAAGGTGAAGGTCGGAGTC-3′ and 5′-GAAGATGGTGATGGGATTTC-3′ (GAPDH). Each sample was tested in triplicate and the differences in cDNA amount were equalized by expression of the housekeeping gene GAPDH.

### Assessment of caspase-7 and-8

Following experimental treatments, the MCF-7 cells were subjected to caspase-7 and-8 activity analysis using caspase calorimetric assay kit (Bio-Vision, Selangor, Malaysia). Briefly, the treated cells were resuspended in 50 μl of chilled cell lysis buffer and incubated on ice for 10 min. The cells were centrifuged for 1 min. at 10,000 × *g* and the supernatant was analysed for caspase-7 and-8 activity suggested by manufacturer protocol. The absorbance was measured at 405 nm in a microtitre plate reader (Bio-Rad).

### Assessment of mitochondrial membrane potential (Δψ_m_)

The measurement of mitochondrial membrane potentials (Δψm) was performed according to the manufacturer instructions (BD MitoScreen, Hercules, CA, USA). Briefly, MCF-7 cells were washed and resuspended in medium at a concentration of 1 × 10^6^ cells/ml. After overnight incubation, the cells were treated with DHA and TAM at the indicated time-points. The cells were incubated with JC-1 (10 μM) at 37°C for another 30 min. Thereafter, cells were washed in PBS and underwent flow cytometry analysis (525 nm). The ratio of Δψ_m_/mitochondrial mass was calculated to correct Δψ_m_ for differences in mitochondrial mass.

### Data analysis

All data were expressed as mean ± SEM unless stated otherwise. Statistical comparisons were made by anova, and *post hoc* Newman–Keuls; differences of *P* < 0.05 were considered significant.

## Results

### Kinetic of vitamin C uptake in MCF-7 cells

**Fig 1:**
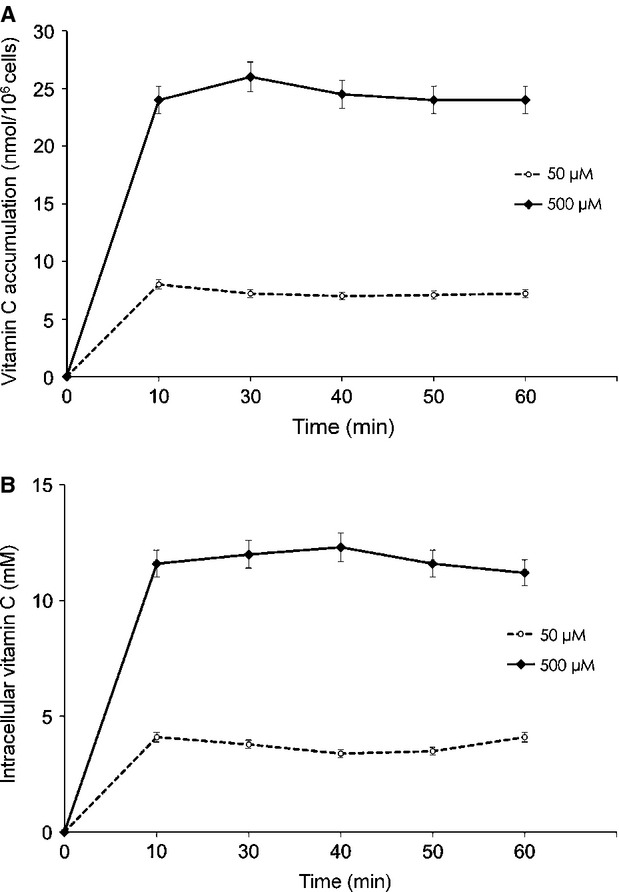
(A) Accumulation of vitamin C in MCF-7 cells after exposure to 50 μM and 500 μM dehydroxy ascorbic acid (DHA). (B) Time-and dose-dependent uptake after exposure to DHA. Results represent means ± SD of at least six independent experiments.

The MCF-7 cells were treated with DHA. As expected, the cells accumulated substantial intracellular concentrations of ascorbic acid in a time-and dose-dependent manner (Fig. 1A and B). The kinetics of DHA uptake was typical of other cell lines 14,24. All cells have glucose transporters and take up DHA that is trapped intracellularly by reduction and accumulated as ascorbic acid 25,26. As indicated in Figure 1, MCF-7 cells accumulated vitamin C within 10 min. Intracellular vitamin C concentrations were dependent on the amount of extracellular DHA offered in the culture medium and rapidly reached a plateau level.

### Vitamin C protects MCF-7 cells from the cytotoxic effect of TAM

**Fig 2:**
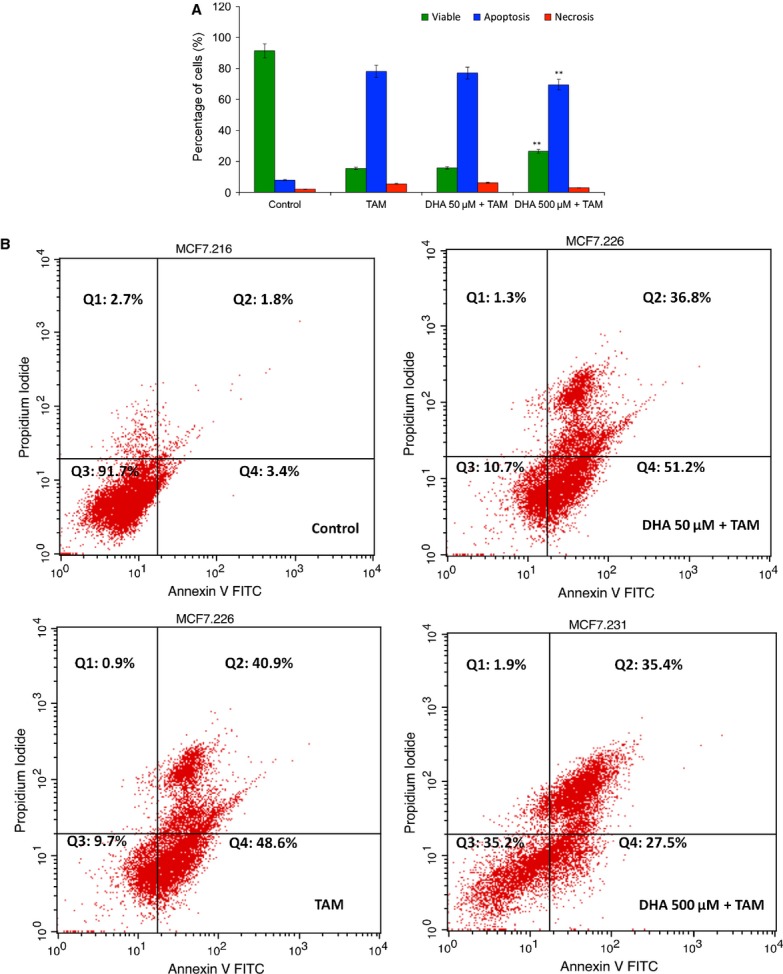
Vitamin C attenuates the cytotoxicity of tamoxifen (TAM) in MCF-7 cells. (A) MCF-7 cells were loaded with dehydroxy ascorbic acid (DHA) to varying concentration of intracellular vitamin C. The cells were washed and treated with TAM for 24 hrs. Morphological assessment of MCF-7 cells stained with AO/PI. The population of viable, apoptotic and necrotic cells was plotted after treatment. (B) MCF-7 cells loaded with various concentration of vitamin C and apoptosis were measured by annexin V/PI staining. Three separate experiments were conducted in triplicate and the results shown represent the mean values and SDs of single representative experiment.

To test whether vitamin C exposure could protect cells from TAM-induced cytotoxicity, the cells were exposed to DHA to increase intracellular ascorbic acid levels, washed and subsequently treated with TAM. AO and PI dyes were used to differentiate viable, apoptotic and necrotic cells under fluorescence microscope. Figure 2A shows the population of viable cells, apoptotic and necrotic cells after the MCF-7 cells were treated with the TAM at IC_50_ concentration for 24 hrs. The apoptotic event of TAM-treated MCF-7 cells was decreased significantly in vitamin C pre-treated cells compared with vitamin C-untreated MCF-7 cells. A small fraction of necrotic cells was also detected in the treatment group but the percentage was negligible. To determine the influence of vitamin C on TAM-associated apoptosis, we performed Annexin V-FITC/PI binding assay, which evaluates phosphatidylserine turnover from the inner to the outer lipid layer of the plasma membrane, an event typically associated with apoptosis. Flow cytometric analysis revealed that the percentage of apoptotic cells with Annexin V-positive but PI-negative cells increased gradually with concentration in TAM-treated cells whereas vitamin C pre-treated MCF-7 cells were decreased in apoptosis. As shown in Figure 2B, after 24 hrs treatment with TAM at IC_50_ concentrations, the numbers of apoptotic MCF-7 cells decreased in 500 μM DHA-treated cells than untreated cells, indicating the pro-apoptotic activity of TAM was decreased. In TAM-treated cells, the proportion of MCF-7 cells in the early apoptosis was 48.6% after 24 hrs (*P* < 0.05 respective to control cells at the same time). For cells that were in the late apoptosis was 40.9% at 24 hrs. Meanwhile, the proportion of the cells in the necrosis was 0.9 at 24 hrs (*P* < 0.05 respective to control cells at the same time). For 500 μg DHA pre-treated MCF-7 cells, the proportion of early and late apoptosis events was reduced to 27.5% and 35.1% respectively (Fig. 2C). Cytotoxicity was reduced more in cells that had higher intracellular concentrations of vitamin C, indicating a dose-dependent effect. High intracellular concentrations of vitamin C, however, had no effect on either the viability or proliferation of untreated cells. This indicates that the protection conferred by vitamin C treatment extends to cells with *in vitro* clonogenic potential. These results also show that vitamin C can attenuate the cytotoxicity of TAM in MCF-7 breast cancer cells and reduces the effectiveness of TAM.

### Vitamin C reduces TAM-caused increase of FasL and TNF-α mRNA expression

**Fig 3:**
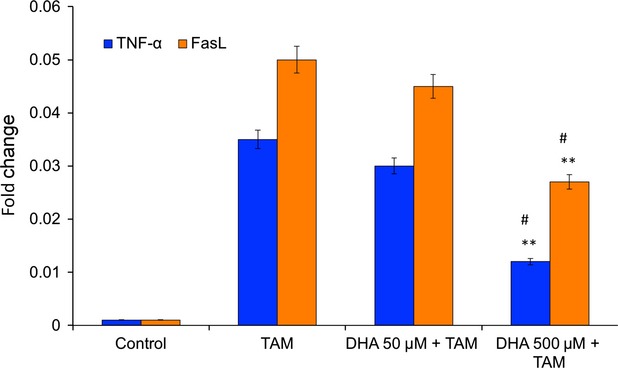
Effect of tamoxifen (TAM) on FasL and TNF-α mRNA expression. The highest amount of FasL and TNF-α mRNA expression were detected after 24 hrs. The influence of different concentration of vitamin C on FasL and TNF-α mRNA expression was analysed. Results represent means ± SD of at least five independent experiments. **P < 0.001 *versus* control; #*P* < 0.01 *versus* TAM.

Significant increases in the amounts of FasL and TNF-α mRNA expression were found in TAM-treated cells compared with control cells (Fig. 3). In comparison to control cells, a 54-fold increase in FasL mRNA expression and a 351-fold increase in TNF-α mRNA expression were assessed. Therefore, the influence of vitamin C on FasL and TNF-α mRNA expression was evaluated after TAM treatment for 24 hrs. A clear decrease in mRNA expression of both factors could be demonstrated by pre-incubating the cells with vitamin C. The percentage of reduction in FasL mRNA expression for MCF-7 cells pre-treated with vitamin C compared with TAM-treated cells 46.4% (500 μM). The same was true for the expression of TNF-α mRNA, which could be reduced by 67.5% (Fig. 3).

### Vitamin C quenches lipid peroxidation and SOD induced by TAM

**Fig 4:**
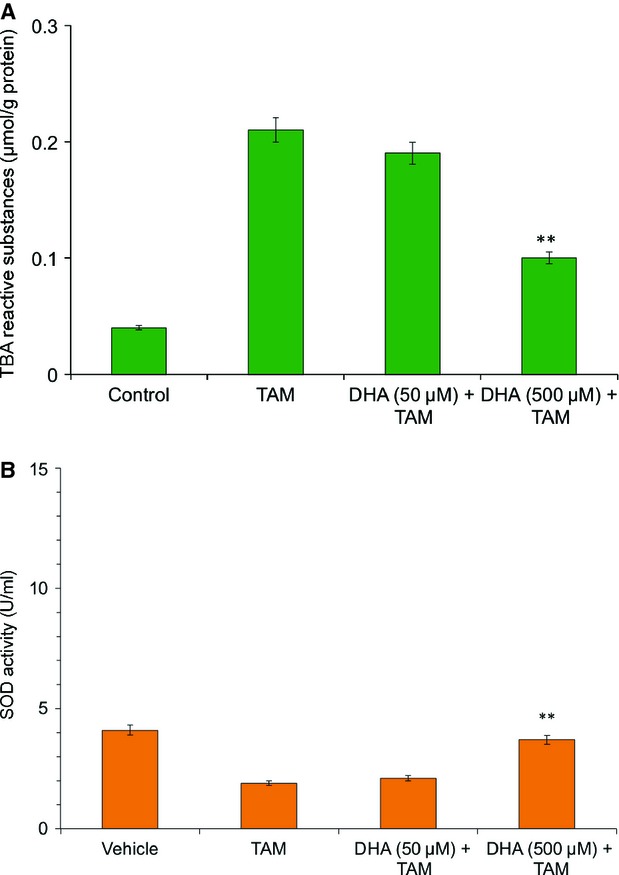
Effect of vitamin C on (A) lipid peroxidation (B) synthesis of superoxide dismutase (SOD). Both the processes were significantly modulated by vitamin C. Values were assessed 24 hrs after tamoxifen treatment. Results represent means ± SD of at least six independent experiments. **P < 0.05.

Vitamin C is a potent antioxidant and quenches ROS in cells under oxidative stress 27,28. To investigate whether vitamin C is able to reduce TAM-generated oxidative cell damage, we measured its influence on lipid peroxidation and SOD. Significant decrease in the amounts of SOD and increase in TBARS were found in MCF-7 cells after TAM treatment compared with control cells. Both processes were significantly altered in a dose-dependent manner by vitamin C (Fig. 4A and B).

### Vitamin C reduces mitochondrial damage induced by TAM

**Fig 5:**
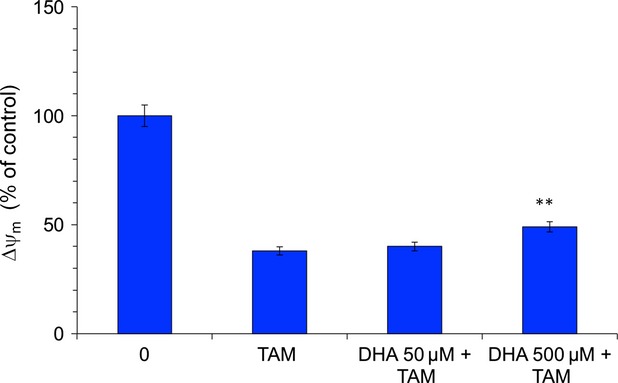
Effect of vitamin C on mitochondrial transmembrane potential was assessed after tamoxifen (TAM) treatment. The Δψ_m_ levels of cells, exposed as units of mean fluorescence intensity, were calculated as percentage of control. Mean ± SD of at least six independent experiments. **P < 0.01 *versus* TAM.

In apoptotic cells, the decrease in mitochondrial membrane differential potential occurs before chromatin condensation and DNA fragmentation 29, and precedes the enhanced release of ROS by the mitochondria 30. As vitamin C prevented the increase in cellular ROS levels induced by TAM, we studied the effect of vitamin C on mitochondrial apoptosis in MCF-7 cells cultured with TAM. We found that loading the cells with vitamin C conferred protection from TAM-induced mitochondrial damage, and these cells maintained high membrane potential (Fig. 5). The TAM-treated MCF-7 cells showed a marked decrease in mitochondrial Δψm compared with control cells but the Δψm was increased in vitamin C pre-treated cells (Fig. 5). These results were consistent with the notion that vitamin C exerts its inhibitory effects against TAM by protecting cells from mitochondrial membrane depolarization.

### Vitamin C reduces TAM-induced caspase-7 and-8 activation

**Fig 6:**
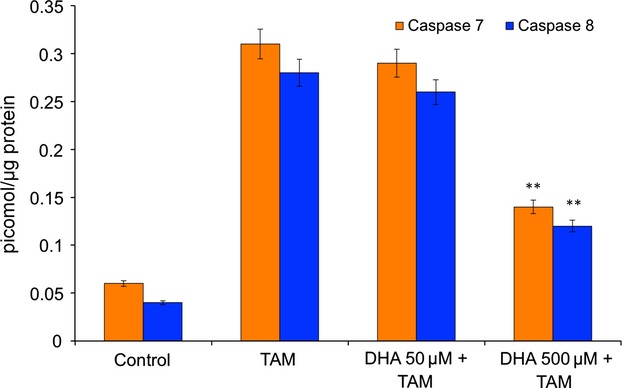
Effect of vitamin C on caspase-7,-8. MCF-7 cells were loaded with various concentrations of vitamin C prior to treatment of tamoxifen (TAM) were lysed and the activity of caspase-7,-8 in the cell lysate were determined. The data represented the mean ± SD of three identical experiments made in the five replicates. **P < 0.01 *versus* TAM.

A consequence of mitochondrial damage during apoptosis is activation of caspase cascade 31, and we studied the effect of vitamin C on the activation of caspase-7 and-8. Caspase-8 is activated upstream in the apoptosis cascade 32. The activation of caspase-7 and-8 was increased after incubation with TAM and was reduced by vitamin C pre-treated MCF-7 cells (Fig. 6).

## Discussion

Many cancer patients take vitamin supplements and lot of surveys reported that the majority of the cancer patients used vitamins combined with conventional therapy 33. The finding that vitamin C antagonized the cytotoxic effects of TAM was unexpected. Vitamin C circulates in the plasma as ascorbic acid; however, it is generally transported into cells as DHA through the facilitative glucose transporters 13,14. Inside the cell, DHA is rapidly reduced to ascorbic acid 14,34. In this way, cells can accumulate high intracellular concentrations of vitamin C. Ascorbic acid is often used in *in vitro* experiments, leading to confounding results because of the lack of direct transport of ascorbate and because ascorbic acid acts as a pro-oxidant in the presence of the free transition metals ubiquitously found in tissue culture 14. We therefore used DHA to load cells with vitamin C 19 and analysed its effect on the intracellular events mediating TAM-induced apoptosis in MCF-7 breast cancer cells. The MCF-7 cells utilized in this study can efficiently transport DHA and accumulate millimolar concentrations of intracellular ascorbic acid. It has been reported that in transformed B cells from chronic lymphocytic leukaemia patients, the intracellular concentration of ascorbic acid is as high as 15 mM 35. How cells *in vivo* acquire vitamin C physiologically is an apparent paradox, as the plasma concentrations of DHA do not exceed 1–2% of ascorbate concentrations 36. However, ascorbic acid can be converted to DHA at the level of the cellular membrane and therefore transported through glucose transporters by the action of superoxide 37. In our study, we focused on the effect of vitamin C on ROS-mediated cancer therapy. Tamoxifen is the recommended drug of choice for breast cancer.

The role of antioxidants in TAM is only marginally examined and very few studies have been published to date concerning antioxidants in TAM treatment. Once DHA is available, the higher uptake by transformed cells compared to normal counter parts 38, increasing their resistance to pro-oxidant agents and chemotherapeutic drugs. Langemann *et al*. found that ascorbic acid in the epithelial cells was significantly higher in neoplastic breast tissue than in non-neoplastic tissue from the same patient 39. The mean increase in ascorbic acid was 2.7 times higher in cancerous tissue 39. A direct comparison of the vitamin C contents of tumour tissues found in the literature and breast cancer cells loaded with 50 μmol/l and 500 μmol/l DHA showed that in our procedures the intracellular levels of ascorbate are absolutely comparable to the *in vivo* situation 23,39. In our study, cells loaded with vitamin C in the form of DHA were significantly protected from TAM-mediated cytotoxicity (Fig. 2). Increasing concentrations of intracellular vitamin C in MCF-7 cells provided increasing protection against lipid peroxidation (Fig. 4A) and SOD generation (Fig. 4B), maintaining the integrity of the mitochondrial membrane (Fig. 5) and increasing the cell viability (Fig. 2). Further downstream effects like the release of caspase-7 and-8 could be diminished by vitamin C (Fig. 6) resulting in a significantly increased survival rate after tumour treatment. Early apoptotic events such as the increased expression of FasL and TNF-α after TAM treatment were significantly reduced by vitamin C (Fig. 3). Besides its modulatory properties of ER-dependent gene transcription, TAM stimulate cholesterol epoxidation and generate cholesterol 5,6-epoxide through ROS control the cancer cell growth 8. Previous studies have reported that antioxidant vitamin E stimulates a full cytoprotection by blocking cholesterol epoxidation induced by TAM in MCF-7 cells 16,17. In this regard to the mechanism of action, it has been shown that autoxidation of intracellular sterols was completely inhibited by vitamin E and furthermore, the macroautophagy induced by anti-oestrogen-binding site ligands was associated with cell survival and protected cancer cells against TAM-induced cell differentiation and death 9,40,41. On the other hand, we demonstrated that vitamin C inhibits the induction of cell differentiation and death by TAM through scavenging ROS production, and speculate that this could be because of impaired 5,6-epoxide cholesterol production. Earlier studies have also shown that cisplatin, doxorubicin, etoposide and several other agents result in mitochondrial membrane depolarization 42,43. We have examined the anti-neoplastic agents TAM that lead to rapid mitochondrial membrane depolarization and have found that this depolarization can be inhibited by vitamin C. Although treatment with anti-neoplastic agents does not generally lead to increased intracellular ROS, it is possible that vitamin C in the mitochondria plays a role in quenching local ROS. Alternatively, vitamin C in the mitochondria may help to stabilize respiratory electron transport, thereby preserving the most efficient means of energy generation and maintaining overall cellular fitness. Taken together, our data show that pharmacological concentrations of intracellular vitamin C antagonize the therapeutic cytotoxic effects of TAM. The increasing intracellular concentrations of vitamin C in cultured MCF-7 breast cancer cells contribute to the resistance to pro-oxidant treatment modalities and may therefore be contraindicated in patients undergoing tumour treatment. This finding could have important clinical relevance, given the wide use of vitamin C as a nutritional supplement. These results suggest that supplementary vitamin C may have adverse consequences in patients who are receiving cancer chemotherapy. Such a supposition, however, would need confirmation in other therapeutic models or in human trials.

## Supporting information

Data S1 Vitamin C attenuates the cytotoxicity of tamoxifen (TAM) in MBA-MD-231 and 4T1 cells.
