# Supplementary material for: Vitamin C suppresses cell death in MCF-7 human breast cancer cells induced by tamoxifen
Source: J Cell Mol Med. 2013 Nov 25;18(2):305–13. doi: 10.1111/jcmm.12188 (PMC3930417; doi:10.1111/jcmm.12188)
Supplement: Data S1 — Vitamin C attenuates the cytotoxicity of tamoxifen (TAM) in MBA-MD-231 and 4T1 cells. [file jcmm0018-0305-sd1.doc]

Dear Prof,

Greetings.

Thank you so much for your valuable comments. We analyzed MDA-MB-231 and 4T1 cells for initial cytotoxicity assay to study the effect of vitamin C and please find the following data. Rest of the experiments were carried out with only MCF-7 cells.

Once again thank you for the kind consideration.

Regards,

Dr. Tamilselvan Subramani.
